# Tracking sub-clonal *TP53* mutated tumor cells in human metastatic renal cell carcinoma

**DOI:** 10.18632/oncotarget.4220

**Published:** 2015-05-20

**Authors:** Guilhem Bousquet, Morad El Bouchtaoui, Christophe Leboeuf, Maxime Battistella, Mariana Varna, Irmine Ferreira, Louis-François Plassa, Diaddin Hamdan, Philippe Bertheau, Jean-Paul Feugeas, Diane Damotte, Anne Janin

**Affiliations:** ^1^ Université Paris Diderot, Sorbonne Paris Cité, Laboratoire de Pathologie, Paris, France; ^2^ INSERM, Paris, France; ^3^ AP-HP-Hôpital Saint-Louis, Service d'Oncologie Médicale, Paris, France; ^4^ AP-HP-Hôpital Saint-Louis, Service de Pathologie, Paris, France; ^5^ Centre Hospitalier de Marne-la-Vallée, Service d'Oncologie Médicale, Jossigny, France; ^6^ INSERM, Paris, France; ^7^ AP-HP-Hôtel-Dieu, Service de Pathologie, Paris, France

**Keywords:** metastases, human RCC, tumor heterogeneity, sub-clonal tumor cells, TP53 mutation

## Abstract

Renal Cell Carcinomas (RCCs) are heterogeneous tumors with late acquisition of *TP53* abnormalities during their evolution. They harbor *TP53* abnormalities in their metastases. We aimed to study *TP53* gene alterations in tissue samples from primary and metastatic RCCs in 36 patients followed up over a median of 4.2 years, and in xenografted issued from primary RCCs.

In 36 primary RCCs systematically xenografted in mice, and in biopsies of metastases performed whenever possible during patient follow-up, we studied p53-expressing tumor cells and *TP53* gene abnormalities.

We identified *TP53* gene alterations in primary tumors, metastases and xenografts.

Quantification of tumors cells with *TP53* gene alterations showed a significant increase in the metastases compared to the primary RCCs, and, strikingly, the xenografts were similar to the metastases and not to the primary RCCs from which they were derived.

Using laser-microdissection of p53-expressing tumor cells, we identified *TP53*-mutated tumor cells in the xenografts derived from the primary RCC, and in a lung metastasis later developed in one patient. The mutation enabled us to track back their origin to a minority sub-clone in the primary heterogeneous RCC.

Combining *in situ* and molecular analyses, we demonstrated a clonal expansion in a living patient with metastatic RCC.

## INTRODUCTION

Metastatic renal cell carcinoma (RCC) is a severe disease with median survival of between 7 and 30 months [1, 2]. Twenty-five percent of patients with localized RCC develop metastases, but clinical and biological factors predicting metastatic risk are not reliable enough to guide RCC therapies at a localized stage. This could be linked to heterogeneity of the tumor cells, recently characterized in primary RCCs [3, 4, 5].

According to the clonal evolution model, oncogenic selection increases the phenotypic and genotypic heterogeneity of cancer cells within tumors [6, 7]. A malignant tumor is composed of sub-clones, and additional mutations can occur during evolution [8]. In RCC carcinogenesis, mutations of the *Von Hippel Lindau* (*VHL*) and *PBRM1* genes are early events [3, 5], and additional mutations, including in *TP53* tumor suppressor gene, are found in metastatic samples [3]. In a whole-exome analysis of 106 primary RCCs, *TP53* mutations have been found in less than 5% of cases [5]. Their frequency may be underestimated, since *TP53* mutations are frequently sub-clonal in primary RCCs [4]. In addition, p53 expression is associated with an increased risk of metastases [9, 10]. Recently, on metastatic samples from the autopsy of a patient with prostate cancer, the lethal metastatic clone had genomic alterations, including a *TP53* mutation, which enabled to track its origin back to a minority sub-clone with the same *TP53* mutation in the primary tumor [11].

In living patients, a comparison between primary tumors and metastases, required to detect such minority sub-clones able to metastasize, is difficult to realize. Xenografts from human cancer tissue are currently used as pre-clinical models [12, 13]. In RCCs, the engraftment rate for primary RCCs with metastatic disease at diagnosis is higher than for localized primary RCCs [14]. This makes this pre-clinical model suitable for biological studies of metastatic RCC.

Here, in 36 patients with RCC followed up over a median of 4.2 years with a systematic xenograft of their primary tumor, we compared p53-expressing cells in tissue samples of the primary RCCs, their corresponding metastases when available, and the xenografts if the engraftment was successful.

## RESULTS

### p53 expression in primary RCCs and in corresponding metastasis

**Figure 1:**
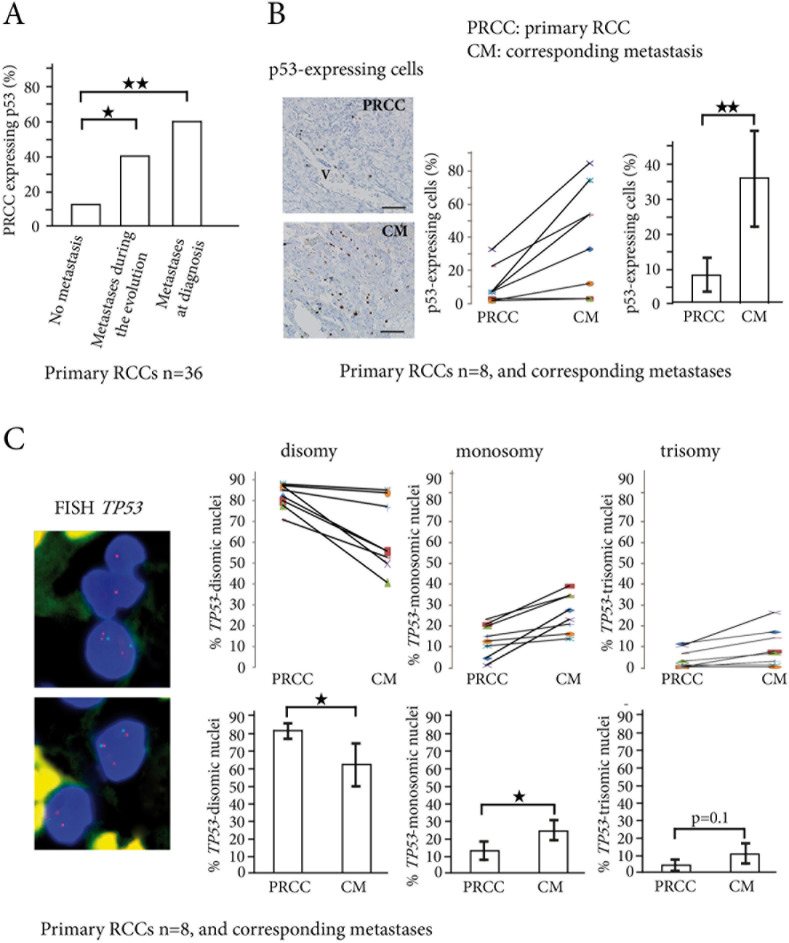
p53-expressing tumor cells and TP53 copy number abnormalities in primary RCCs and corresponding metastases **A.** In the 36 primary RCCs, the percentage of tumors expressing p53 (threshold >1% p53-positive cells) significantly increases from the primary RCCs (PRCC) with no metastasis to the PRCCs with metastases during evolution or at diagnosis. **B.** p53-expressing cells are found around vessels (V) in primary RCC, and are scattered in the corresponding lung metastasis. Indirect immunoperoxydase, bar =50μm. In 8 patients with biopsy samples of the primary RCCs (PRCC) and their corresponding metastases (CM), the percentage of p53-expressing cells significantly increases in the metastases. **C.** *TP53* gene copy number abnormalities are assessed by FISH in the same 8 patients, with *TP53* gene labeled in red and chromosome 17 centromere in green. Nuclei are colored in blue by DAPI. The upper panel shows one trisomic nucleus (three red spots) and two monosomic nuclei (one red spot in each) for *TP53* gene. The lower panel shows one trisomic cell and one disomic cell. Counts of TP53 gene copy numbers show a significant decrease in the percentage of disomic cells in the metastases, while the percentage of monosomic cells significantly increases. *p < 0.05, **p < 0.01.

In 36 primary RCCs we first assessed p53 expression using immunohistochemistry. A minimum number of 5 tumor blocks were tested for each RCC, and a threshold of more than 1% p53-expressing cells was used, according to Uhlman et *al*. [10]. In the 180 blocks analyzed from the 36 primary RCCs, 15/180 (8%) were positive for p53 staining. When the 36 primary RCCs with 5 blocks tested were considered, 13/36 (36%) of them expressed p53, a result in accordance with a previous *in situ* study [10]. When we only considered the 21 patients with localized RCC at diagnosis, the percentage expressing p53 was significantly higher for the patients who later developed metastases than for the patients who did not (40% vs 12.5%). In the other 15 that had metastasis at diagnosis, 60% were expressing p53. The numbers of p53-expressing cells were thus larger in patients with metastasis, either at diagnosis or during the metastatic evolution, than in patients with no metastasis (Figure 1A and Supplementary Table 1). The sensitivity of p53 positive staining on primary RCCs for predicting metastasis was 55%, with a predictive positive value of 84.6%. When we calculated the Chi-square between these two parameters, we found that p53 expression on primary RCC was linked to metastatic risk (X² = 6.96, *p* < 0.01).

We then focused on the 8 metastatic patients with available biopsy samples of their metastasis (Patients 25 to 28 and 33 to 36, in Table 1). When the mean numbers of p53-expressing cells were compared in the 8 primary RCCs and in their corresponding metastases, they were significantly larger in the metastatic samples (from 8% to 36%, *p* < 0.01) (Figure 1B). Using FISH for *TP*53 on the same samples (Figure 1C and Supplementary Table 2), we found a significant increase in abnormalities of *TP53* gene copy numbers when primary RCCs were compared to their corresponding metastases (17.8% *vs.* 34.7%, *p* < 0.01). *TP53* monosomic tumor nuclei were found in 13.6% *vs.* 24.7% (*p* < 0.05), and *TP53* trisomic tumor nuclei in 4.2% *vs.* 10% (*p* = 0.1), when primary RCCs were compared to their corresponding metastases (Figure 1C).

**Table 1:** Patient characteristics and RCC engraftment in mice

Patients	Age/ gender	Primary RCC (PRCC)			Corresponding metastasis (CM)		
		TNM (25)	Fürhman grade	Engraftment of PRCC	Metastasis	Metastasis biopsy	Time of Metastasis
1	49/M	pT1bN0M0	2	No	No		
2	69/F	pT3bN0M0	3	No	No		
3	46/M	pT3bN0M0	3	No	No		
4	57/M	pT1bN0M0	3	No	No		
5	62/M	pT1bN0M0	3	No	No		
6	72/M	pT1aNxM0	3	No	No		
7	58/M	pT1bNxM0	2	No	No		
8	57/M	pT1aNxM0	3	No	No		
9	66/M	pT1aN0M0	3	No	No		
10	72/M	pT1aNxM0	2	No	No		
11	59/M	pT1aNxM0	3	No	No		
12	68/M	pT3aNxM0	2	No	No		
13	83/F	pT1bN0M0	3	No	No		
14	58/M	pT1bN0M0	2	No	No		
15	67/M	pT1aNxM0	3	No	No		
16	80/F	pT2N0M0	2	No	No		
17	70/F	pT3aNxM0	2	No	Yes	No	During the evolution
18	58/M	pT3aN0M0	4	No	Yes	No	During the evolution
19	67/M	pT4N1M0	4	No	Yes	No	During the evolution
20	59/F	pT3aNxM0	3	No	Yes	No	During the evolution
21	76/M	pT1bN0M1	3	No	Yes	No	At diagnosis
22	81/F	pT3aNxM1	3	No	Yes	No	At diagnosis
23	45/M	pT3N0M1	2	No	Yes	No	At diagnosis
24	71/M	pT3bNxM1	3	No	Yes	No	At diagnosis
25	54/M	pT3bNxM1	3	No	Yes	Yes	At diagnosis
26	62/M	pT3bN0M1	3	No	Yes	Yes	At diagnosis
27	55/M	pT2N0M1	3	No	Yes	Yes	At diagnosis
28	63/M	pT3aNxM1	3	No	Yes	Yes	At diagnosis
29	68/M	pT4NxM1	4	Yes	Yes	No	At diagnosis
30	62/F	pT3aN1M1	3	Yes	Yes	No	At diagnosis
31	51/F	pT3bN1M1	3	Yes	Yes	No	At diagnosis
32	52/M	pT3bN1M1	4	Yes	Yes	No	At diagnosis
33	63/M	pT1aN1M1	4	Yes	Yes	Yes	At diagnosis
34	57/M	pT3bNxM1	4	Yes	Yes	Yes	At diagnosis
35	67/F	pT3bN1M1	4	Yes	Yes	Yes	At diagnosis
36	74/M	pT1aNxM0	3	Yes	Yes	Yes	During the evolution

Taken together, these results are in favor of an expansion of tumor cells bearing *TP53* gene alterations from the primary stage to the metastatic stage of human RCCs.

### p53 expression in primary RCCs, derived xenografts and corresponding metastases

For the 36 primary RCCs followed up over a median period of 4.2 years, we systematically performed xenografts at the time of initial surgery. Successful engraftment occurred in 8 cases (Patients 29 to 36, in Table 1). The engraftment rate was significantly higher in the 15 RCCs with metastatic disease at diagnosis than in the 21 localized RCCs (47% *vs*. 5%, *p* < 0.01).

In the 8 cases with successful engraftment, tumor samples from the primary RCC, the corresponding metastasis and the xenograft were available for 4 patients (Patients 33 to 36, see Table 1). For two of these, there was no p53-expressing cell in any sample.

**Figure 2:**
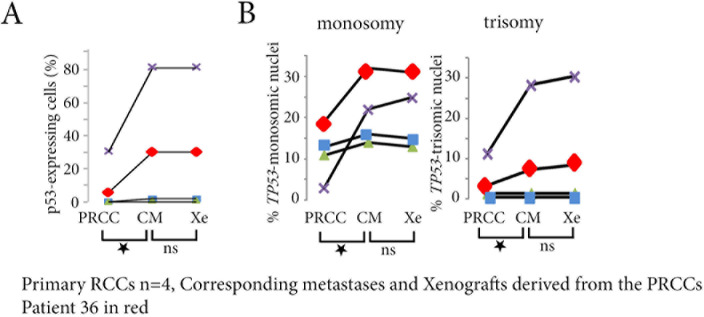
p53-expressing tumor cells and TP53 copy number abnormalities in primary RCCs, corresponding metastases and xenografts derived from the primary RCC **A.** In the 4 patients with biopsy samples of the primary RCCs (PRCC), of their corresponding metastases (CM), and of the xenografts (Xe), the percentage of p53-expressing cells significantly increases in the metastases and in the xenografts for Patient 35 (in purple) and for Patient 36 (in red), without any difference between the metastasis and the xenograft of a same patient. **B.** In the same 2 patients, the xenograft is reflecting the metastasis, and both are different from the primary RCC, with increased numbers of *TP53* monosomy and trisomy in the corresponding metastasis and in the xenograft.ns = not significant, *p < 0.05.

In the other two patients (35 and 36), we first compared the primary RCCs to the xenografts derived from them. The numbers of p53-expressing cells (Figure 2A), and the percentage of tumor cells with *TP53* gene copy number abnormalities (Figure 2B), were significantly larger in the xenografts (*p* < 0.05 for both). We then compared the xenografts and patient metastases. There was no significant difference in the numbers of p53-expressing cells (Figure 2A) or in the percentage of *TP53* gene copy number abnormalities (Figure 2B).

Taken together, these results show that the numbers of tumor cells with *TP53* gene alterations increase similarly in xenografts derived from primary RCCs and in metastases that developed later in the same patients.

### Transcriptomic analyses of the *TP53* pathway

Frozen biopsy samples of the primary RCC, the corresponding metastasis, and the xenografts were available for Patient 36, and trancriptomic analyses were performed on them. When we clustered the lung metastasis and the two xenografts, and compared them to the primary RCC, 468 genes were differentially expressed (fold change> 2, *p*-value< 0.05, data submitted online to Gene Expression Omnibus, Supplementary Excel File 1). Using qRTPCR, we validated these results for four of the most differentially expressed genes *ADAMTS12, PYHIN1, RSPO4* and *RAB3A* (Supplementary Figure 2).

**Figure 3:**
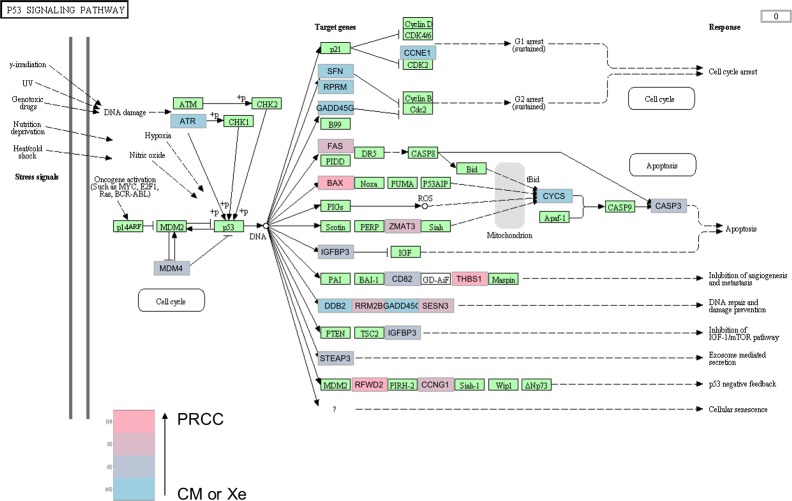
*TP53* signaling pathway in the lung metastasis and the two xenografts compared to the primary RCC of patient 36 In tumor samples from patient 36, the *TP53* cell signaling pathway is significantly altered in the metastasis and in the xenografts compared to the primary tumor, with 22 genes differentially expressed. The fold changes are indicated with a color gradient from blue (primary tumor) to pink (metastasis or xenografts). The fold changes are indicated with a color gradient from blue (metastasis or xenografts, CM or Xe) to pink (primary tumor, PRCC). When a gene is significantly overexpressed in the xenografts and in the lung metastasis, the box is colored blue (for example *ATR*). When significantly overexpressed in the primary tumor, the box is colored pink (for example *BAX*).

The pathway analysis showed that 4 cell signalling pathways were significantly altered in the metastasis and in the xenografts when compared to the primary RCC (Table 2). The *TP53* pathway had 22 genes differentially expressed (Figure 3, Supplementary Excel File 2).

**Table 2:** Patient 36, cell pathways altered on transcriptomic analyses comparing primary RCC with corresponding metastasis and the two xenografts derived from primary RCC

Cell signaling pathway	Number of genes with expression change	*p*-value
GnRH	28	0.09
Cytosolic DNA	17	0.05
MAPK	70	0.04
**p53**	**22**	**0.04**

### Tracking sub-clonal *TP53*-mutated tumor cells

**Figure 4:**
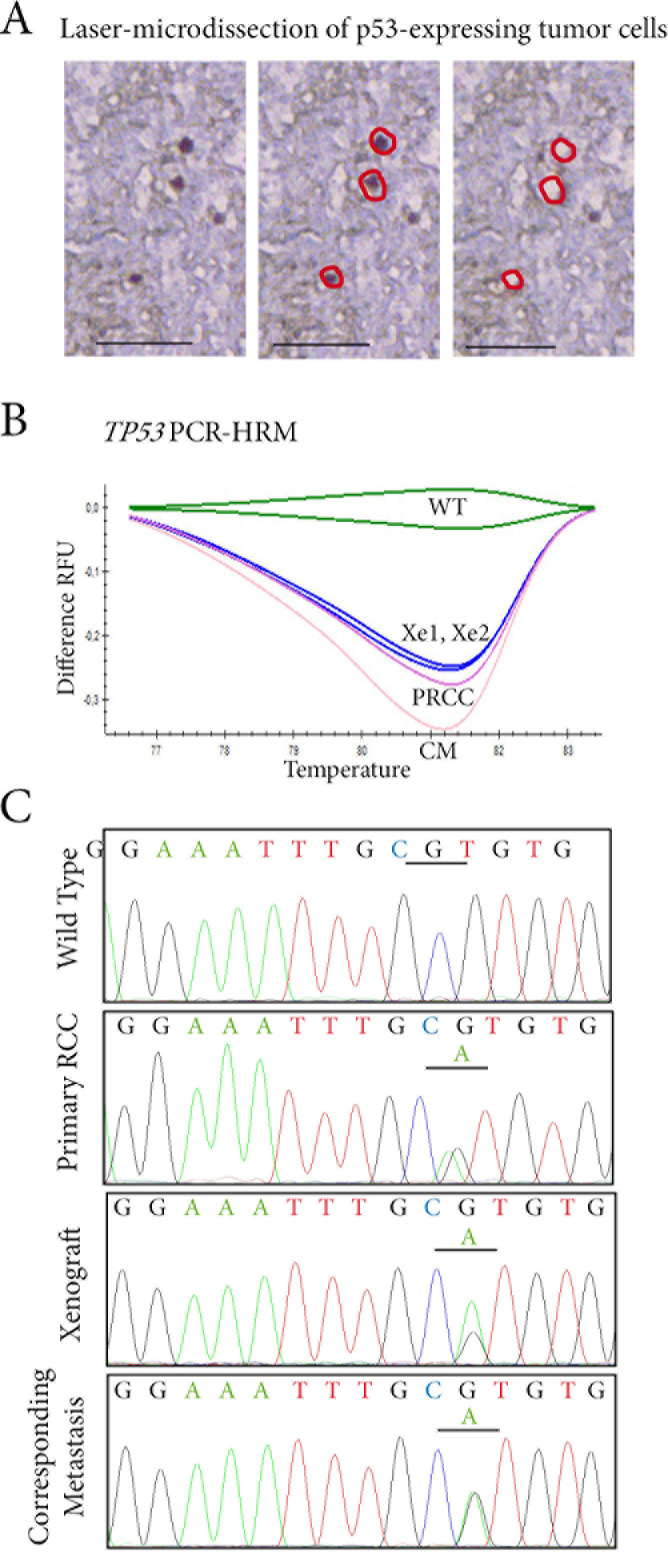
Tracking *TP53* mutated tumor cells in p53 expressing cells by laser-microdissection **A.** In the RCC lung metastasis sample, p53-expressing cells are laser-microdissected for further molecular analyses. **B.** *TP53* status is assessed by PCR-HRM screening on exons 5 to 8 in p53-expressing cells microdissected from the primary RCC (PRCC), the two xenografts derived from it (Xe1, Xe2), and the lung metastasis (CM). A shift on exon 6 is identified in the four samples when compared to wild-type profile (WT). **C.** Sequencing of exon 6 of *TP53* identifies an identical missense mutation c.605G>A p.R202H in laser-microdissected p53-expressing cells from the primary RCC, in the two tumor xenografts derived from it, and in the lung metastasis.

To decipher the links between primary RCCs, their corresponding metastases, and xenografts at cellular level, we decided to look for *TP53* mutations in p53-expressing cells. Since p53-expressing cells were few and scattered in the tumor samples, we performed a laser-microdissection of these p53-expressing cells (Figure 4A). Using PCR-HRM to screen for *TP53* exons 5 to 8, one patient (Patient 36) had a mutated profile. For the other patient (Patient 35), the HRM profile was similar to the wild-type profile.

For patient 35, we further assessed microsatellite profiles for *TP53* on the three different tumor samples. We identified a LOH (loss of heterozygosity) in the xenograft derived from the primary RCC. When we analyzed allelic imbalances, we showed a decrease in the allelic peak ratio from the normal tissue of the patient to the primary RCC and the metastasis (Supplementary Figure 1).

At the time of surgery and RCC xenograft, Patient 36 had a localized RCC at low risk for relapse [15]. However, two xenograft models Xe1 and Xe2 were obtained from this localized primary RCC. Follow-up of this patient demonstrated that in fact he was at a pre-metastatic stage, since he developed lung metastasis six months after initial surgery. This clinical situation enabled us to study the primary RCC at a pre-metastatic stage, the xenograft, and the surgically removed metastasis.

Using PCR-HRM, we found a similar *TP53-*mutated profile with a shift on exon 6 in the primary RCC, in the two xenografts derived from it and in the lung metastasis (Figure 4B). This was not the case for p53-negative cells, which had a PCR-HRM profile for *TP53* similar to the wild-type profile in the four samples.

Further sequencing of *TP53* gene exon 6 identified a missense mutation c.605G>A p.R202H in p53-microdissected cells from the lung metastasis. An identical missense mutation was found in the two xenografts and also in the primary RCC (Figure 4C).

Taken together, these results show that we were able, in a living patient, to track *TP53* mutated tumor cells in a pre-metastatic RCC, in the metastasis and in two xenografts derived from the primary RCC.

## DISCUSSION

We focused this study on *TP53* abnormalities in tissue samples obtained in the evolution of primary and metastatic RCCs in 36 patients followed up over a median of 4.2 years.

As soon as 1994, p53 expression in primary RCCs has been significantly associated with an increased risk of metastases. [10] Using the threshold established for p53 positivity in this study, we found that p53 expression in the 36 primary RCCs was higher in patients with metastases either at diagnosis or during evolution.

We compared the primary RCCs and their corresponding metastases when samples were available (8 patients), and found an increase not only in p53-expressing tumor cells, but also in *TP53* gene abnormalities *in situ* in metastatic samples. These results are in accordance with those of Gerlinger *et al.* who performed exome sequencing, chromosome aberration analysis, and ploïdy profiling on tumor samples of 4 primary RCCs and corresponding metastases. They found *TP53* mutations only in metastatic samples while *VHL* and *PBRM1* mutations were early carcinogenetic events [3].

To better decipher the links between primary RCCs and their corresponding metastases at cellular level, we performed laser microdissection to select p53-expressing cells and study their mutational profile for *TP53*. In the metastatic sample of one patient, we demonstrated the expansion of a *TP53* mutated clone which was a minority in the primary RCC. These results in a human are original and complementary to the cartographic and genomic study by Gerlinger *et al*. [3] who proposed the concept of branched evolutionary tumor growth. Our dynamic study in a living patient reinforces this concept and the clonal evolution theory [6, 7]. However, the fact that we identified a minority *TP53*-mutated clone in the primary tumor later expanded in the metastasis suggests that molecular abnormalities responsible for the metastatic process are usually not highlighted by whole tissue analyses of primary tumors. In addition, in a case of prostate cancer, a majority lethal *TP53*-mutated clone, identified from autopsy metastatic samples using whole-exome sequencing, was also a minority in the primary tumor [11]. These findings and ours emphasize the importance of performing studies combining *in situ* analyses of protein or gene expression and molecular analyses.

Our results also open discussion about the role of p53 in the RCC metastatic process. p53 directly controls the transcription of genes implicated in metastatic evolution [16]. In experimental mouse models of the Li-Fraumeni syndrome, *TP53* mutations, different from the c.605G>A p.R202H *TP53* mutation we identified, have been shown to increase the incidence of metastases [17]. The mutation we identified has not been previously described in RCC, although it is located on exon 6, a preferential location for RCC *TP53* mutations (http://p53.iarc.fr) [18, 19].

Xenografts had been systematically performed at the time of initial surgery in the 36 primary RCCs in our study. In the patient with *TP53* mutation, the mutated sub-clone also expanded in the xenografts. This unexpected result led us to compare the data obtained in the xenografts and in the metastases *versus* the primary RCCs. We found similar results for xenografts and metastases, both significantly different from primary RCC results, for the numbers of p53-expressing tumor cells and for *TP53* molecular genetic abnormalities detected *in situ* and on transcriptomic analyses. If the expansion of a minority clone between a primary tumor and the corresponding metastasis was expected, even if it is particularly difficult to demonstrate *in vivo* in a patient, the similarities between xenografts derived from a primary tumor and a metastasis later developed in the same patient are surprising results, with real translational potential.

Recently, Ding *et al*., using whole-exome sequencing, demonstrated similarities between xenografts and metastases in a highly aggressive breast cancer [20].

The engraftment rate in mice is itself higher in aggressive RCCs, particularly those with metastases [14]. This was also the case for our 36 primary RCCs, and, strikingly, the only non-metastatic RCC at diagnosis with a successful engraftment later developed lung metastases. Our study and that by Ding *et al*. are in favor of a selection, through the engraftment mechanism, of the most aggressive clones which are able to drive a metastatic process. These clones, which can be a minority in the primary heterogeneous RCC, are precisely those that should be detected early, because they expand in the metastases with their own molecular characteristics, different from the majority clones in the primary RCC. The metastatic clones need to be tested to optimize drug efficiency, and xenografts are pre-clinical models suitable for therapeutic tests.

This systematic study of 36 RCCs with biopsies of metastases and xenografts of the primary RCC reflects the difficulty of studying metastatic evolution in humans, even if numerous new cellular and molecular technologies are now available. It also shows the high interest of analyzing metastatic samples whenever possible, since the expansion of minority clones implies that drug sensitivity will differ between the primary tumor and the metastases.

The fact that the xenografts are closer to the metastases than to the primary tumors opens fields for future research in the area of metastatic disease, with possible prediction of metastatic risk when engraftment is successful, and discussion of clinical trials using individual xenografts for personalized treatment of metastatic patients [21]. The feasibility is real, since engraftment is rapid (time-lapse under six weeks when the median progression-free survival is 41 weeks for metastatic RCC treated first-line with sunitinib [2]) and seems linked to metastatic potential. For the research centers with animal facilities and close collaboration between clinicians and researchers, patient-derived xenografts could be an additional innovative tool to improve metastatic cancer therapy, in RCCs or in other uncontrolled malignant tumors.

## MATERIALS AND METHODS

### Patients with RCC

Between 2006 and 2012, we performed xenografts of fresh tumor samples from 36 patients with clear-cell RCCs. Patients and tumor characteristics are summarized in Table 1. There were 27 men and 9 women, and the median age of the patients was 62.5 years (range, 45-83 years).

At diagnosis, 21 patients had localized RCC and 15 had metastatic disease. After a median follow-up of 4.2 years, among the 21 patients with localized RCC, 5 secondarily developed metastases (Patients 17 to 20, and Patient 36). Biopsy samples from metastatic sites were available for one of these five patients (Patient 36). For the 15 other patients with metastases at diagnosis, biopsy samples from metastatic sites were available for 7 patients (Patients 25 to 28, and 33 to 35).

### RCC xenografts

Sub-cutaneous xenografts of fresh samples from human clear-cell RCCs were performed in nude mice. Samples were obtained from primary tumors, before any medical treatment. All the samples were similarly processed and cut into three parts: one was formaldehyde-fixed and paraffin-embedded, one was snap-frozen in liquid nitrogen and stored in Hôpital-Saint-Louis Tumorbank, and one was put in culture medium for xenografting in nude mice.

In compliance with French Bioethics-law (2004-800, 06/08/2004), all patients had been informed of the research use of the part of their samples remaining after diagnosis had been established, and did not oppose it. Informed consent was obtained from each patient. The University Institute Board Ethics Committee for experimental animal studies approved this study (N°2012-15/728-0115).

### Detection of p53-expressing tumor cells

For all samples, an indirect immunoperoxidase method using anti-human p53 mouse antibody (clone DO7, Dako, Glostrup, Danemark) as primary antibody was performed on 5μm-thick tissue sections. The secondary antibody was a rabbit monoclonal anti-mouse IgG1 H&L (clone M1gG51-4, Abcam, UK) coupled with antirabbit OmniMap detection kit (Roche diagnostic, Meylan, France). The systematic controls used were absence of primary antibody and use of an irrelevant primary antibody of the same isotype.

For all tissue sections, p53-expressing cells were counted on five different fields at x400 magnification. A ProvisAX70 microscope (Olympus, Tokyo) with wide-field eyepiece number 26.5 was used, providing a field size of 0.344mm^2^ at x400 magnification. Only stained nuclei of tumor cells were considered positive. For each field, 100 tumor cells were analyzed. The percentage of p53-expressing cells was the number of positive cells among these 100 tumor cells. Results were expressed as mean ± standard error of the mean (SEM).

A minimum number of 5 tumor blocks were tested for each of the 36 primary RCCs. For one patient, we retained the maximum percentage value of p53-expressing cells among the 5 tissue sections and 25 fields analyzed. We could thus compare matched samples: five blocks for a primary RCC, one block for the corresponding metastasis and one or two blocks for xenografts derived from the primary RCC.

### *TP53* gene copy-number alterations

Fluorescent *in situ* hybridization (FISH) for *TP53* with TexasRed-labeled 17p13.1 (*TP53* gene) probe and FITC-labeled CEP17 (chromosome 17 centromere) probe (p53 kit, Cytocell-Aquarius, Cambridge, UK) was performed on 7μm-thick tissue sections of 8 primary RCCs and of 8 metastatic tumor samples corresponding to the 8 primary RCCs. Slides were processed using the Histology FISH accessory kit® (Dako, Denmark) according to the manufacturer's recommendations. The slides were hybridized overnight with the specific *TP53* probe. Samples were analyzed with an AxioImager.M1 epifluorescence microscope (Zeiss, Hamburg, Germany). Images were captured with a x63 oil immersion objective and were analyzed using the Isis® software (METAsystems, Altlussheim, Germany). At least 200 intact, non-overlapping nuclei were scored for each sample. The numbers of *TP53* alleles (red fluorescence) and CEP17 (green fluorescence) were recorded in each cell, to determine the prevalence of each genetic abnormality. The percentage of *TP53* copy-numbers (monosomy, disomy or trisomy) was the number of nuclei with 1, 2 or 3 *TP53* alleles among the 200 tumor nuclei analyzed. Results were expressed as mean ± standard error of the mean (SEM).

### Allelic profile analyses of *TP53*

For Patient 35, laser-microdissection of p53-expressing tumor cells was performed on 7μm-thick tissue sections from the primary RCC, from the xenograft obtained from it, and from the peritoneal metastasis. A minimum number of 1000 p53-expressing tumor cells were microdissected for each sample, for TP53 gene analysis. Total DNA was extracted from microdissected cells using DNeasy-Mini-Kit (Qiagen, Les-Ulis, France), quantified on NanoDrop and qualified by electrophoresis.

Polymerase Chain Reaction (PCR) was performed using 10 ng DNA for each PCR. Characteristics of the two microsatellite dinucleotide repeat markers are detailed in Supplementary Table 3. The PCR mix contained 1 U Taq Gold (Applied Biosystems, Foster City, CA, USA), 2.5–4 mM MgCl2, 0.2 mM dNTP, 0.2 μM labelled forward primers (NED™, FAM (6-carboxyfluorescein) or VIC™) and 0.2 μM non-labelled reverse primers. The PCR final volume was 20 μl. Thirty-five cycles of PCR were performed. After denaturation, the PCR products were run on ABI PRISM 310 Genetic Analyser. The analysis of the migration data was performed with Genescan 3.1 software (Applied Biosystems).

The fluorescent allelic profiles obtained from microdissected tumors and DNA from normal tissue from the same patient were compared. All these profiles were verified in two different experiments. The ratio of allelic peaks was calculated for each sample, enabling the measurement of allelic imbalances. Loss of heterozygosity (LOH) was defined as loss of one tumor allele.

### TP53 mutational status analysis

Laser-microdissection of p53-expressing tumor cells was performed on 7μm-thick tissue sections from the primary RCC, from the two xenografts obtained from it, and from the lung metastasis. Using a PALM-Microbeam/Zeiss-system on tissue sections immunostained with anti-human p53 antibody, a minimum number of 1000 p53-expressing tumor cells were microdissected for each sample, for *TP53* gene analysis. As controls, 1000 p53-negative tumor cells were also laser-microdissected for each sample. Total DNA was extracted from microdissected cells using DNeasy-Mini-Kit (Qiagen, Les-Ulis, France), quantified on NanoDrop and qualified by electrophoresis.

PCR-High Resolution Melting (PCR-HRM) was performed using primers designed by NCBI-Reference-SequenceX54156 (see Supplementary Table 4, according to Bastien *et al.*, Ref. 23), and synthesized by Eurogentec. PCR was carried out on the CFX96^TM^ Real Time System (Bio Rad, Hercule, California) on a total volume of 20μL containing 5μl of genomic DNA, 10μL of SsoFast^TM^Evagreen supermix 2X (Bio Rad) and 0.4μM of both forward and reverse primers. PCR was performed with an initial denaturing step at 94°C for 2 mn, followed by 45 cycles of denaturing (95°C for 5 s), and annealing (60°C for 10 s). A post-amplification melting curve program was initiated by heating to 95°C for 1mn, cooling to 50°C for 1mn, and continuously increasing the temperature by 0.2°C to finally reach 95°C. Post-amplification fluorescent melting curves were analyzed with the Bio-Rad Precision Melt Analysis software (Bio Rad) [22]. Each PCR run included a no-template control, and each sample was run in triplicate.

Following this PCR-HRM screening of laser-microdissected cells, sequencing of the shift fragment for exon 6 of *TP53* was performed using the Sanger method [23]. Amplicons 80 to 150bp-long covered the coding sequence and exon-intron boundaries. All forward primers were tailed with M13-Universal nucleotidic sequence for sequencing standardization. For sequencing, 20μL of PCR products were purified using ExoSAP-IT product cleanup (USB Corporation, Cleveland, USA). Labelling was performed using BigDye®-Terminator-v1.1 Sequencing-Kit (Applied-Biosystems, Foster-City.CA, USA) in both forward and reverse. The reaction was run according to the following protocol: an initial denaturing step at 94°C for 3 mn; 25 cycles at 94°C for 10 s, annealing temperature at 60°C for 20 s. Purified products were run on a 16-capillary automated sequencer (ABI-PRISM®-3130xl-Genetic-Analyzer, Applied-Biosystems, Foster-City, CA, USA). SeqScape-Software v 2.5 (Applied-Biosystems, Foster-City, CA, USA) enabled nucleotide change determination.

### Gene-expression profiling and *TP53* pathway

Total RNA was extracted from the frozen tumor sample using RNeasy-Mini-Kit (Qiagen, France), quantified on NanoDrop and qualified on Bio-Rad Experion^TM^ Automated-Electrophoresis-Station (BioRad, France). Transcriptomic analyses were performed using MiltenyiBiotec Microarray service. A linear T7-based amplification step was performed from 0.5 μg of all RNA samples. To produce Cy3-labeled cRNA, the RNA samples were amplified and labeled using Agilent-Quick-labeling kit. The yields of cRNA and the dye-incorporation rate were measured with ND-1000 Spectrophotometer (NanoDrop, LabTech, France). Hybridization was performed according to the Agilent 60-mer oligo-microarray processing protocol: 1.65 μg Cy3-labeled cRNA was hybridized overnight at 65°C to Agilent-Whole-Human-Genome-Oligo-Microarrays 4×44K, and fluorescence signals were detected using Agilent's Microarray-Scanner. Agilent-FE-Software determined feature intensities, and quantile normalization was performed with the Agi4×44PreProcess R package. Differential expression between transcriptomes was analyzed with R 3.01 software (Foundation for Statistical Computing, Vienna, Austria) and based on log2 single-intensity expression data. Pathway analysis was carried out with DAVID (Database for Annotation, Visualization and Integrated Discovery, http://david.abcc.ncifcrf.gov) Bioinformatics Resources 6.7 [24] and the “KEGG profile” (Kyoto Encyclopedia of Genes and Genomes) R package. We indicated the number of genes with expression change in each pathway and the p-value corresponding to a modified Fisher exact p-value similar to a hyper-geometric test. p-values smaller than 0.05 usually correspond to strongly enriched categories. The DAVID database does not use q-values since q-values are dependent on the cut-off chosen to define the differentially expressed genes.

Using qPCR, we validated transcriptomic analyses for the four following human genes: *ADAMTS12* [Hs00229594_m1], *PYHIN1* [Hs02385967_m1], *RSPO4* [Hs01382765_m1] and *RAB3A* [Hs00623221_m1]. Total RNA was reverse-transcribed (cDNA) before qPCR amplification using random primers with SuperScript TM II Reverse Transcriptase (Invitrogen, France). The qPCR reactions were performed using fluorescent probes on a CFX96 Real Time System (Bio-Rad). A blank sample (no cDNA) was included and the experiments were performed in triplicate for each gene, each sample being in duplicate on the PCR plate. The housekeeping gene *TBP* [Hs99999910_m1] was used to normalize gene expression results. The results were expressed as 2^−ΔΔCT^ (also called relative quantification, RQ).

### Statistical analyses

Calculations were carried out using SPSS Statistics 17.0 software or R 3.01 statistical software. For counts of p53-expressing tumor cells, the mean ±SEM was calculated in each tumor sample (primary RCC, metastasis or tumor-xenograft), and displayed in bar graphs.

Quantitative values were compared using Student's t-test (two-tailed). P values under 0.05 were considered significant.

## SUPPLEMENTARY FIGURES AND TABLES






