# Supplementary material for: Tracking sub-clonal TP53 mutated tumor cells in human metastatic renal cell carcinoma
Source: Oncotarget. 2015 May 20;6(22):19279–89. doi: 10.18632/oncotarget.4220 (PMC4662490; doi:10.18632/oncotarget.4220)
Supplement: Supplementary file 1 [file oncotarget-06-19279-s001.pdf]

Tracking sub-clonal *TP53* mutated tumor cells in human metastatic renal cell carcinoma

Supplementary Material

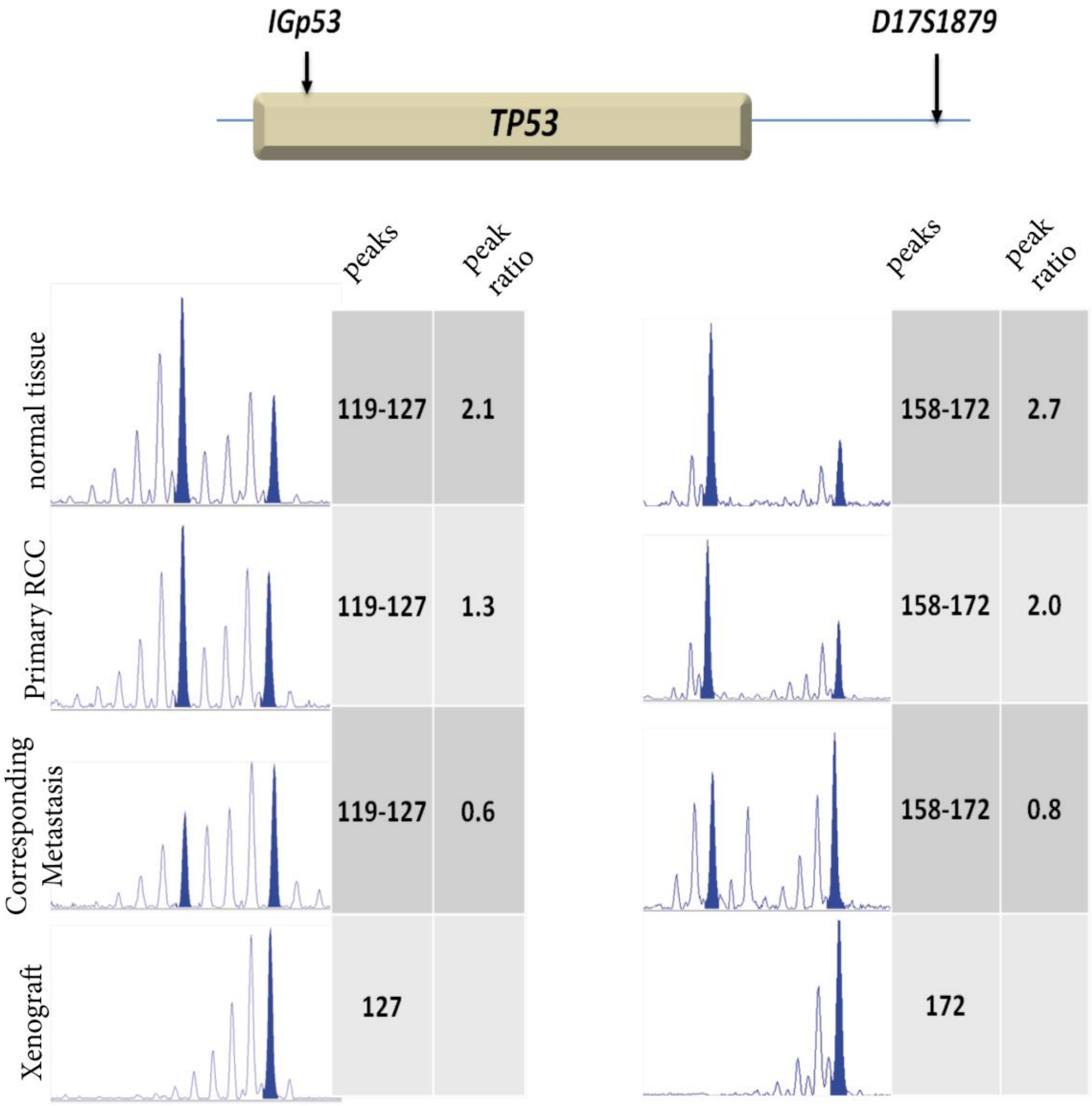

Supplementary Figure 1

Allelic profile analyses of *TP53* were performed for Patient 35 on the following tissue samples: the normal tissue, the primary RCC, the corresponding peritoneal metastasis, and the tumor xenograft derived from the primary RCC.

Left panel shows allelic profiles for the microsatellite marker IGp53, with two peaks at 119 and 127 bp in the normal tissue. There is a loss of the first peak in the xenograft defining a

LOH (loss of heterozygoty). A similar profile is observed for the second microsatellite marker D17S1879 (right panel). For the two markers, there is also a decrease in the allelic peak ratio from the normal tissue of the patient to the primary RCC and the corresponding metastasis.

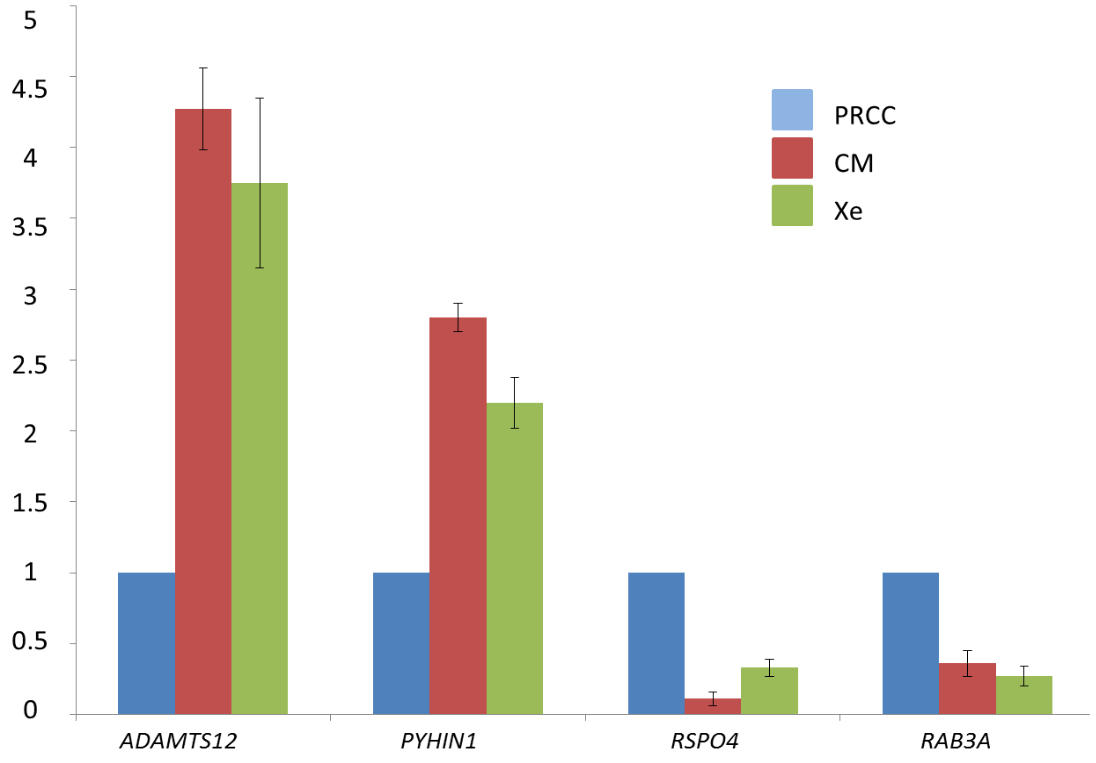

Supplementary Figure 2

qRTPCR was performed on the same tumor samples that have been used for transcriptomic analyses for Patient 36: the primary RCC (PRCC), the corresponding metastasis (CM), and the xenografts (Xe) derived from the PRCC. The experiments were performed in triplicate. The housekeeping gene *TBP* was used to normalize gene expression results. The results are expressed as $2^{-\Delta\Delta CT}$ (also called relative quantification, RQ), and show an increased mRNA expression of *ADAMTS12* (RQ=4.2 and 3.7) and *PYHIN1* (RQ=2.8 and 2.2) in the CM and in the Xe compared to the PRCC, and a decreased mRNA expression of *RSPO4* (RQ=0.1 and 0.3) and *RAB3A* (RQ=0.4 and 0.3) in the CM and in the Xe compared to the PRCC.

Supplementary Table 1: % of p53-expressing tumor cells on multiple tumor blocks

Patients	**Primary RCC (PRCC)**					**Corresponding Metastasis (CM)**
	B1	B2	B3	B4	B5	B
1	0	0	0	0	0	-
2	0	0	0	0	0	-
3	5	0	0	0	0	-
4	0	0	0	0	0	-
5	0	0	0	0	0	-
6	0	0	0	0	0	-
7	0	0	0	0	0	-
8	0	0	0	0	0	-
9	0	0	0	0	0	-
10	0	0	0	0	0	-
11	0	0	0	0	0	-
12	0	10	5	0	0	-
13	0	0	0	0	0	-
14	0	0	0	0	0	-
15	0	0	0	0	0	-
16	0	0	0	0	0	-
17	0	0	0	0	0	-
18	0	0	0	0	0	-
19	10	0	0	0	0	-
20	0	0	0	0	0	-
21	0	5	0	0	0	-
22	60	0	0	0	30	-
23	0	0	0	0	0	-
24	0	0	0	0	5	-
25	0	5	0	0	0	70
26	20	0	0	0	0	50
27	5	0	0	0	0	50
28	0	0	0	0	0	10
29	5	0	0	0	0	-
30	0	5	5	0	0	-
31	0	0	0	0	0	-
32	0	0	0	0	0	-
33	0	0	0	0	0	0
34	0	0	0	0	0	0
35	0	0	30	10	0	80
36	0	5	0	0	0	30

B1 = Block 1

Supplementary Table 2: % of *TP53* abnormalities on FISH on multiple tumor blocks

patients	Primary RCC (PRCC)										Corresponding Metastasis (CM)	
	B1		B2		B3		B3		B5		B	
	M	T	M	T	M	T	M	T	M	T	M	T
1	-	-	-	-	-	-	-	-	-	-	-	-
2	-	-	-	-	-	-	-	-	-	-	-	-
3	18	3	10	0	13	0	10	0	9	0	-	-
4	-	-	-	-	-	-	-	-	-	-	-	-
5	-	-	-	-	-	-	-	-	-	-	-	-
6	-	-	-	-	-	-	-	-	-	-	-	-
7	-	-	-	-	-	-	-	-	-	-	-	-
8	-	-	-	-	-	-	-	-	-	-	-	-
9	-	-	-	-	-	-	-	-	-	-	-	-
10	-	-	-	-	-	-	-	-	-	-	-	-
11	-	-	-	-	-	-	-	-	-	-	-	-
12	11	0	24	0	18	0	11	0	9	0	-	-
13	-	-	-	-	-	-	-	-	-	-	-	-
14	-	-	-	-	-	-	-	-	-	-	-	-
15	-	-	-	-	-	-	-	-	-	-	-	-
16	-	-	-	-	-	-	-	-	-	-	-	-
17	-	-	-	-	-	-	-	-	-	-	-	-
18	-	-	-	-	-	-	-	-	-	-	-	-
19	22	5	12	0	11	0	15	0	11	0	-	-
20	-	-	-	-	-	-	-	-	-	-	-	-
21	9	0	13	3	9	0	11	0	7	0	-	-
22	29	17	11	0	13	0	11	0	20	8	-	-
23	-	-	-	-	-	-	-	-	-	-	-	-
24	11	0	9	0	6	0	10	0	15	4	-	-
25	5	0	6	12	7	0	6	0	5	0	26	18
26	22	7	13	0	15	0	11	0	11	0	32	15
27	20	0	11	0	9	0	7	0	12	0	36	8
28	13	0	18	0	14	0	16	0	15	0	20	3
29	17	4	8	0	13	0	12	0	12	0	-	-
30	11	0	19	0	18	1	9	0	11	0	-	-
31	-	-	-	-	-	-	-	-	-	-	-	-
32	-	-	-	-	-	-	-	-	-	-	-	-
33	11	0	13	0	10	0	12	0	12	0	14	1
34	13	0	12	0	18	0	16	0	13	0	16	0
35	16	0	14	0	3	11	7	9	14	0	22	28
36	9	0	19	3	11	0	12	0	11	0	32	7

B1 = Block 1
M = monosomy, T = Trisomy

Supplementary Table 3: characteristics of the two microsatellite dinucleotide repeat markers used for allelic profile analyses of *TP53* for Patient 35.

Marker	Locus	Heterozygosity index	Size mini	Size maxi	F.PRIMER 5'	R.PRIMER 5'	Gene
IGp53	17p13.1	0.90	97	138	AggATACT ATTCAgCC CgAggTg	gTTTCTTA CTgCCACT CCTTgCCC CATTC	p53 intron 1
D17S1879	17p13.1	0.82	135	173	gAAgTTTA CgAAAATT gCTgCT	gTTTCTTA AgggTAgTT CTgCgTgC	p53 (centro)

Supplementary Table 4: Primer sequences for *TP53* analysis

Exon	Forward sequence	Reverse sequence	Primer set	Position		Amplicon size, bp	Amplicon, CG%
				Forward	Reverse		
5	GCCCTGACTTTCAACTCTGTCT	ACAGCTGCACAGGGCAGGTCTT	X5D1	12325-12348	12403 -12424	100	54.0
	TGCCCTCAACAAGATGTTT	CACAACCTCCGTCATGTG	X5D2	12372-12392	12490 -12507	136	61.0
	GGCCATCTACAAGCAGTCA	CAACCAGCCCTGTCGTCT	X5D3	12468-2488	12568 -12585	118	61 .8
6	CCTCACTGATTGCTCTTAG	GTGTTTCTGTCATCCAAATACT	X6D1	12610 -12627	12682 -12701	92	48.9
	TCTTATCCGAGTGGAAG	CCAGTTGCAAACCAGAC	X6D2	12648 -12666	12742 -12758	111	48.6
7	TGGGCCTGTGTTATCTCCTA	CCCATGCAGGAACTGTTAC	X7D1	13289 -3309	13250 -13368	80	50.0
	TTAGTTGTAACAGTTCCTGCATGG	ATAGATTGGCAAGTGGCTCCTGA	X7D2	13349 -13367	13421-13436	88	58.0
8	TGCCTCTTGCTTCTCTTTTC	CTTTCTTGCGGAGATTCTCTTC	X8D1	13730 -13749	13836 -13857	128	52.4
	GTGTTTGTGCCTGTCCT	CTCCACCGCTTCTTGTC	X8D2	13794 -13812	13912 -13930	137	60.3
